# Shining a light on an additional clinical burden: work-related digital communication survey study – COVID-19 impact on NHS staff wellbeing

**DOI:** 10.1057/s41599-022-01427-7

**Published:** 2022-11-18

**Authors:** Ameet Bakhai, Leah McCauley, Liba Stones, Saria Khalil, Jay Mehta, Nicola Price, Vinodh Krishnamurthy, Lucy H. H. Parker, Derralynn Hughes

**Affiliations:** 1 Royal Free London NHS Foundation Trust, London, UK; 2 Aberdeen Royal Infirmary, London, UK

**Keywords:** Science, technology and society, Social policy, Health humanities

## Abstract

Work-related communication volume within the United Kingdom’s National Health Service (NHS) has had little systematic research previously. The impact of communication volume on work-life balance of healthcare staff in the NHS is also not known and has not been an area of focus or governance. COVID-19 led to a shift to non-physical work, with greater reliance on digital communication for clinical decision making. We sought to elucidate the relationship between communication, work-life balance, and COVID-19. An online survey was conducted to assess the platforms used to communicate professionally, the volume of and time spent on work-related communications, how this has changed from before to during COVID-19, and the effect on work-life balance. A total of 3047 healthcare staff provided consent and evaluable data. Emails were reported as the most frequently used communication tool, and the majority of staff asked, reported increased work-related communications due to COVID-19. Staff estimated receiving 14 emails on an average day before COVID-19. During the pandemic, staff estimated getting approximately 17 emails on an average day and 29 emails on a busy day. Work communications reportedly took up increased amounts of family and home time during COVID-19. A large proportion (36%) of staff were unable to switch off from work-related communications already before COVID-19, worsening (57%) during the pandemic. Work-related digital communication is a vital component of working in the NHS. We provide the first detailed data on the types, volume, and impact of such communication on NHS staff during the COVID-19 pandemic, compared to pre-pandemic levels. We found that 82% of staff support the need for NHS guidance on work-related communications to help manage overload, protect emotional wellbeing, and increase resilience. Further work is urgently needed in this area to tackle the negative impact of communication technologies (technostress) on work-life balance to reduce staff stress, burnout, and turnover or early retirement of some staff.

## Introduction

On 30 January 2020, the World Health Organisation declared a public health emergency of international concern in relation to the COVID-19 outbreak in China (World Health Organisation 2020). The National Health Service (NHS) declared an internal Level 4 serious incident and commenced preparations. On 17 March, all NHS Trusts were instructed by the NHS Chief Executive and Chief Operating Officer to take immediate measures for reducing the spread of the virus and to prepare for an imminent surge of COVID-19 patients. This resulted in an unprecedented shift across all aspects of healthcare services to streamline efforts and optimise management of COVID-19 patients (Belkin et al. 2020; Stevens and Pritchard 2020). For many healthcare staff, this meant a notable overhaul of work patterns, as well as redeployment to emergency rotas in areas of urgent need. Many staff returned to frontline clinical duties from academia, research, or retirement (Blake et al. 2021). Conversely, some staff became unavailable for frontline clinical duties as they were unwell, shielding, or self-isolating. Remote working increased, minimising face-to-face contact with patients, relatives, and other staff (Wang and Low 2019; Hutchings 2020).

Effective communication is a key domain of good clinical practice for doctors, nurses, managers, and all health professionals in the NHS (General Medical Council 2019). Staff in the NHS often work in multidisciplinary or multi-agency teams, communicating with team members, other professionals involved in providing care, and with patients and their relatives or advocates (The King’s Fund 2019; Dettmers et al. 2016; Iliffe 2008). Agile communication is paramount in crisis situations to coordinate a service under pressure, such as tracking patient capacity, coordinating movements of patients, coordinating staffing, or providing staff and leadership briefings (Hutchings et al. 2021; Skryabina et al. 2021). Instant messaging applications, particularly WhatsApp, are common in clinical practice (Morris et al. 2021) and enhance communication during emergencies where rapid coordinated response is vital (Skryabina et al. 2021; Nikolic et al. 2018). Conversely, ineffective communication is associated with low staff morale, poor patient experience, adverse patient outcomes and increases staff stress and burnout rates (Patel et al. 2018; Nohammer and Stichlberger 2019).

Digital communication has been recognised as an important area of ‘technostress’, that is, a cause of workplace stress arising from the digitisation of work (Tarafdar et al., 2014). Excessive email communication has also been noted to be associated with a sense of being overwhelmed, which can lead to sub-optimal self-rated health, cognitive disturbances, and symptoms of burnout (Stich et al. 2018; Puranik et al. 2020, Marsh et al. 2020). Higher email load (Stich et al. 2019) demands to reply rapidly (Becker et al. 2021), and organisational norms and expectations to monitor email in non-work hours (Gadeyne 2018; Waldhauser 2019) have been found to be associated with higher levels of stress. We set out to examine work-related communication and its relationship to the work-life balance of healthcare workers, aiming specifically to determine the impact of the COVID-19 pandemic.

## Methods

### Outline, eligible participants, data processing, informed consent, and regulatory approval

The ‘Communications and COVID-19’ study was designed as an online survey with four sections. Healthcare professionals providing consent were eligible to complete the survey. To confirm the eligibility criteria were met, respondents were asked to confirm they have access to an NHS email account (nhs.net or nhs.uk domains). Respondents who confirmed they have access to an NHS email account were deemed eligible and could continue with the survey.

Participants were informed that all data collected would be maintained in line with the Data Protection Act 2018 and used for the purpose of providing medical insights to improve standards and inform solutions to tackle work-related communications issues. Informed consent -encompassing consent to participate and agreement to data collection, analyses, and publication - was collected in the initial survey questions. As the study involved NHS staff, Health Research Authority (HRA) (20/HRA/2445) approval was applied for and granted to conduct the study in England and Wales. Although the HRA confirmed that participating NHS organisations were not required to formally confirm Capacity and Capability (C&C) to undertake the research, some NHS organisations required C&C confirmation prior to inviting their staff to participate. An application to obtain permission to conduct the study in Scotland was made to NHS Research Scotland (NRS); however, an approval was not granted due to concerns over the survey burden on NHS Scotland staff during the COVID-19 pandemic.

### Survey design

Section 1 of the survey asked respondents for demographics and characteristics, such as gender, age, their work role, the healthcare sector they work in, their working pattern during the COVID-19 pandemic, and their direct contact with patients with suspected COVID-19. The survey included 32 various work roles, as well as an ‘Other’ option with a text field to specify a particular work role not stated. All roles stated in the survey were encompassed by one of the 10 official NHS staff group categories (NHS Digital 2022): Additional Clinical Services, Additional Professional Scientific & Technical, Administrative & Clerical, Allied Health Professionals, Estates & Ancillary, Healthcare Scientists, Management, Medical & Dental, Nursing & Midwifery, and Students.

Section 2 asked respondents to select the communication platforms they used during the COVID-19 pandemic and the frequency of use at their busiest. A specific question regarding the use and understanding of Microsoft Teams was also included, as this communication platform was made available to all NHS email users between 6 and 20 March 2020 to facilitate remote working (Hughes 2020).

Section 3 of the survey examined the volume and time spent on work-related communication, both at work and off-duty, before and during the COVID-19 pandemic.

In Section 4, respondents were asked to rate their perceived ability to manage communications, including their experiences of communication interfering with work, travel, home life, and leisure time, as well as the ability to disconnect when needed. The period referred to as ‘before COVID-19’ was defined in the survey as being ‘the last three months in 2019’. Where respondents were asked to reflect on experiences both before and during COVID-19, this definition enabled comparison of similar timeframes across respondents and provided a means of elucidating how the pandemic impacted staff.

All data capture is via the survey requesting estimates from staff of current work practices and of practices prior to the pandemic in the earlier time period prior to the pandemic. Five-point Likert scales were used in the survey where possible. Examples of scales used include Markedly Increased to Markedly Reduced (with About the same as a neutral category) when identifying how COVID-19 changed work communication volume, and Strongly Agree to Strongly Disagree (with Not Sure as a neutral category) for several questions, including when considering statements pertaining to the volume of communication before and after COVID-19. The survey also asked respondents to consider the frequency of receiving particular types of emails both before and during COVID-19, using a scale of Constantly to Never (including Hourly, Daily, Weekly, and Rarely). A ranking question was also included, which entailed participants dragging and dropping nine types of work communication into their subjective order of usage.

Finally, looking to the end of the pandemic and beyond, we asked for respondents’ views on future trends and changes affecting the delivery of clinical care from 2021 onward. Participants were asked whether they felt guidance or a policy on managing work-related communication would be useful. A question section on WhatsApp was incorporated into the survey on 28 May, a week after the survey launch, when early results indicated the importance of this line of communication during the pandemic.

### External validation of survey data

A request under the Freedom of Information Act (FOIA) 2000 was made to NHS Digital, a body responsible for the operation of NHSmail, to obtain data on the volume of emails sent from/to nhs.uk addresses in the period November 2019 to May 2020. The self-reported volume of emails received by the survey respondents was extrapolated into an estimate of the total monthly email volume received by NHSmail users and compared with the actual total as reported by the NHS Digital FOIA response (NHS Digital 2020), dated 10 July 2020 for the month of November 2019.

### Study development and delivery

Sponsored by the Royal Free London NHS Foundation Trust, the survey was conducted using the QualtricsXM online survey platform (Qualtrics 2022), made available via desktop and optimised for Smartphone access. A secondary website was licenced via the Jisc Research Surveys (Jisc Online Surveys 2022) website for users unable to access the Qualtrics platform for any reason.

The survey opened on 21 May 2020. All NHS organisations included in the National Directory of NHS Research Offices maintained by the NHS Research and Development (R&D) Forum were invited to take part in the study. 10 NHS Trusts issued survey permission and distributed the study invite to their staff, with other organisations still processing permission at the time of survey closure. Some Trusts used an internal internet advert, whilst others included the invitation in regular executive updates to all staff. An invite was also sent to the members of the UK Research and Development Directors (UKRD) forum—a body representing UK NHS R&D leaders.

### Survey sample size, response patterns and interim analysis

The initial sample size of 400 participants was informed by the limited body of research on work-related communication surveys. The sample size was considered significant compared to Barber et al. (2019), who surveyed 254 respondents in their survey of telepressure. However, as further NHS Trusts were issuing permissions and distributing the survey to their staff, the types of centres and roles represented were expanding. Therefore, the survey was kept open to collect additional data and allow the more recently invited staff to participate, such as staff from mental health trusts and ambulance trusts.

After 30 days, an interim analysis using a temporal analysis by tertiles was conducted in order to confirm data saturation. The analysis data to be similar between the first, second and third thousand respondents except for COVID-19 exposure patterns. Over time, data show a slight increase in staff working part time from 18.7 to 21.4%, and remotely from 21.5 to 27.3%. An increase from 19.2 to 25.9% was seen in reported frequency of physical exposure to patients with COVID-19 across the survey period. Other data were stable, and respondents included hospital trusts, community trusts, mental health trusts, and ambulance trusts. Academic locations and GP practices were slightly less represented as these organisations took longer to approve this form of research and share the survey links internally. As further insights were unlikely, and we did not wish to cause further survey distraction to staff during the pandemic, the survey was closed on 19 June 2020.

## Results and analysis

### Survey results

**Fig. 1:**
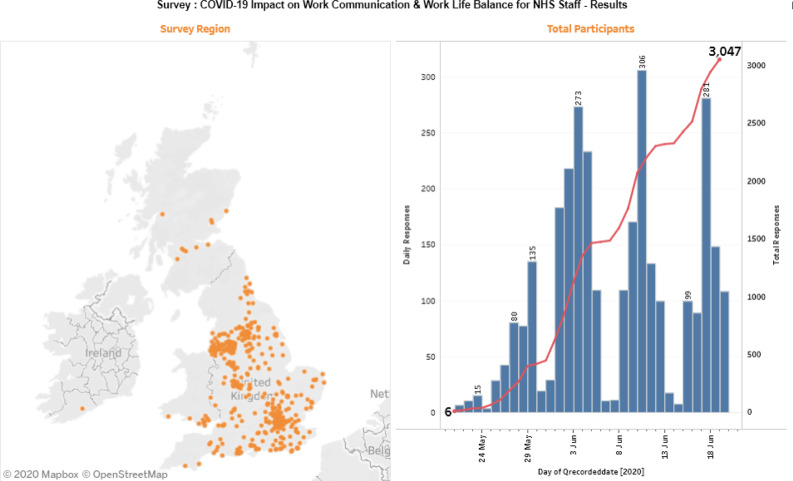
Figure showing survey accruals and geolocation of survey respondents. First panel showing geolocation in orange. Second panel showing accruals in blue and totals in red over the time period.

A total of 3611 respondents initiated the study between 21 May and 19 June 2020. A total of 3047 consenting staff confirmed they held an NHS email address (nhs.net or nhs.uk domain) and provided evaluable survey data. The survey averaged 102 responses per day, peaking at 368 responses on 10 June 2020. There were lower response numbers on weekend days, and peak participation on Wednesdays (as shown in Fig. 1). Response rates for over 2500 respondents were complete to all survey data items, with only 109 respondents having more than 40 of the potential 79 data points empty. Responses to questions regarding WhatsApp use (added to the survey on 28 May) were available from 983 respondents.

### Section 1: Baseline data

The age, gender, and working patterns reported by participating staff show that 85% of respondents (2592 of 3047) were women. Women were somewhat overrepresented compared to the NHS total of 77% (NHS England 2021), likely due to the significantly uneven gender distribution in the NHS workforce, where women make up for example 89% of nurses and health visitors, but only 43% of ambulance staff. The highest concentration of respondents was in the 51–60 years age category (29.24%), which represents the 46% of the NHS workforce that are aged 45–64. A total of 35% of all respondents reported working remotely for either the entirety of the workweek or on select workdays. Around 12% of staff reported being redeployed. Almost half (45%) of all staff were working full-time hours or shifts as usual, with only about one in six (15%) working part-time.

For the analysis of work roles, all jobs—including each response in the text field provided for the ‘Other’ category—were manually categorised according to official NHS staff groups. All 10 categories were represented in the survey results, with most respondents being in Nursing & Midwifery (25%), Administrative & Clerical (22%), and Additional Clinical Services (20%) roles.

Most (79%) of respondents agreed to complete a bonus final section ranking types of communication, and 333 responders providing a commentary in the free text final question. Here, many staff attested to stress from high call volumes as patients’ relatives requested information about their loved ones in hospital. Note, we deliberately avoided asking questions not relevant to the survey, including respondents’ employers’ names, but the location of participant IP addresses (Fig. 1) showed that responders were distributed widely throughout England, with some Scottish staff working on English sites taking part whilst working remotely.

### Section 2: Communication types and frequency

Through a review of literature, social media, and by consulting NHS staff, we determined a range of communication platforms that staff could encounter in their day-to-day duties—from email, Microsoft Teams, NHS library-approved apps such as Hospify and Panda, to commonly available applications not designed for medical use, such as WhatsApp and Twitter (Thomas 2018). Despite NHS data governance policies and training dissuading staff from using WhatsApp and other non-secure technologies (NHS England 2022), it was clear that NHS staff were utilising this ubiquitous tool for many types of queries. WhatsApp was the third most frequently used platform reported with 27% of respondents using it either constantly or hourly during the pandemic, behind only emails—with 84% of staff respondents relying on emails either constantly or hourly—and the phone (36%). NHS-approved instant messaging technologies such as Hospify were occasionally used (5% of staff reported weekly usage during the pandemic).

**Fig. 2:**
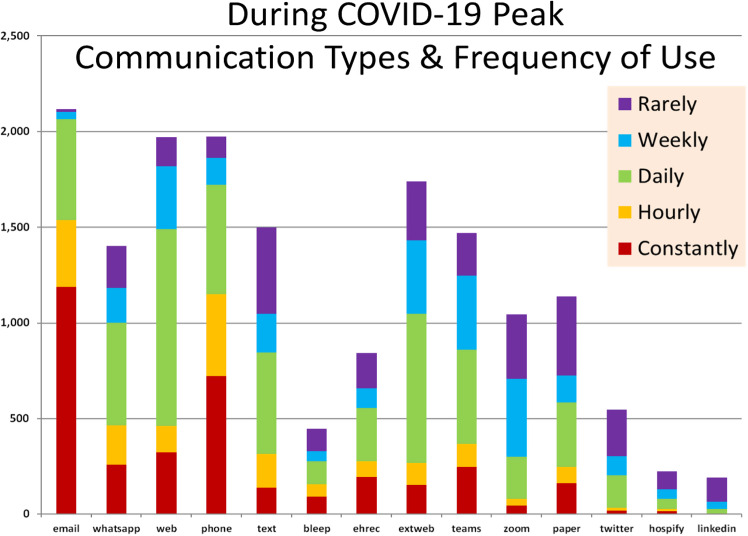
Figure pertaining to data collected from survey Section 2, including the communication platforms reportedly used by participants and frequency of use during COVID-19.

Figure 2 shows the types of communications platforms in use and their frequency of use, as reported at the peak of COVID-19. The electronic healthcare record was less frequently used for work communication than Microsoft Teams and Zoom. Compared to the phone, bleeps appear to be diminishing (with only 2% of respondents reporting weekly usage), but text messaging remains in high use (24% report daily usage), though behind WhatsApp, and far behind email. Email appears unambiguously as the most frequently used platform reported for work-related communications.

### Section 3: Communication volume and the impact of COVID-19

**Fig. 3:**
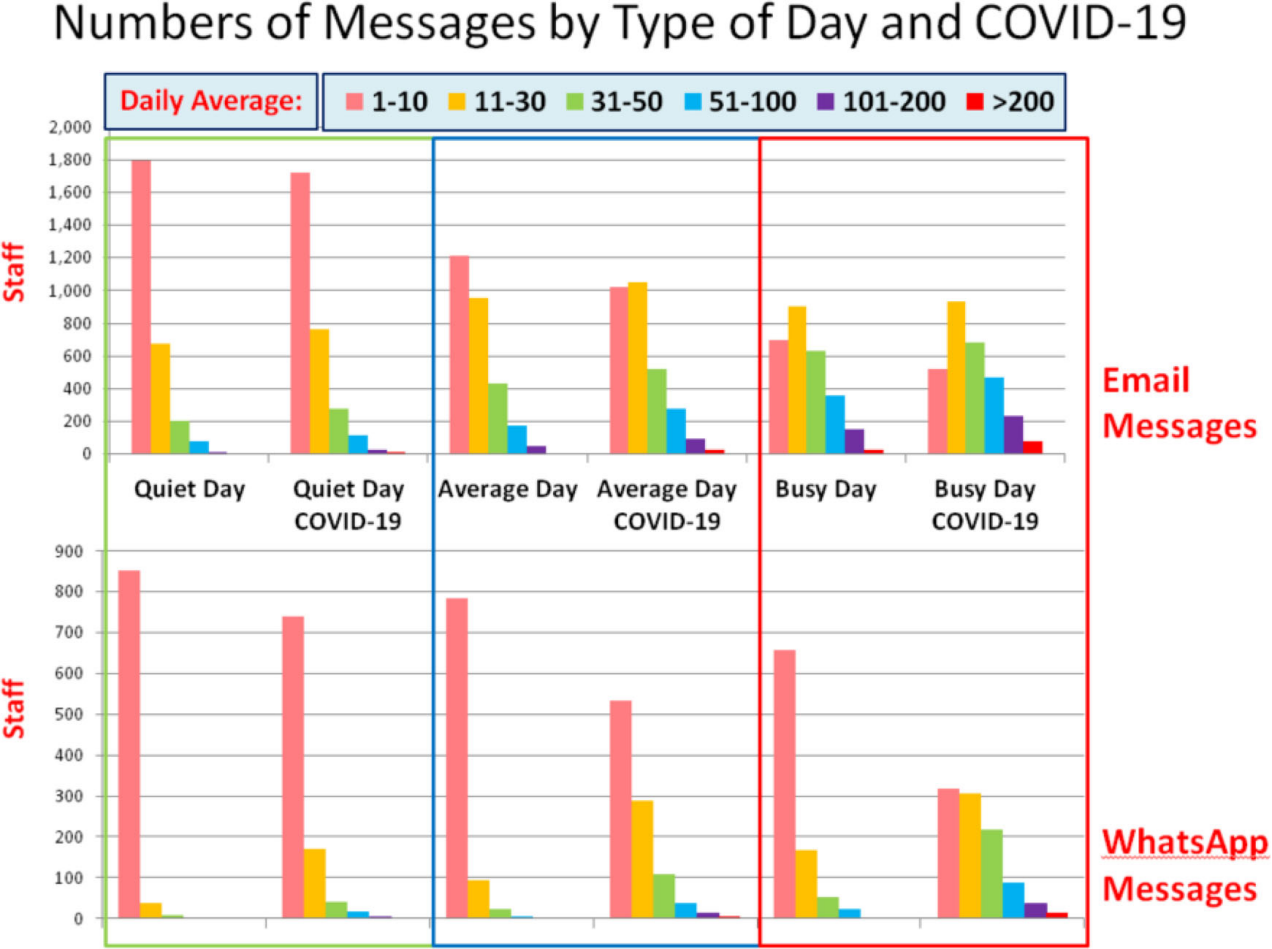
Figure pertaining to data collected from survey Section 2, showing daily average communication volume estimates, before and during COVID-19, by leading platforms—email and WhatsApp.

This section of results relies on respondents’ subjective assessments pertaining to volume of communication. To quantify this, we chose to ask for information about the number of emails and WhatsApp messages as these were the most frequent communication types aside from phone calls. The data relating to these platforms are shown in Fig. 3. In Fig. 3 we see volumes on three different types of days, from a quiet day (such as a Friday or a regular weekend day at work), to an average day (such as a Wednesday), to a busy day (such as a Monday). On a busy workday, 16% of respondents reported receiving between 51 and 100 emails per day prior to the pandemic. This increased to 23% of staff during COVID-19. Overall, staff estimated receiving about 14 emails per day on average days prior to the pandemic, which increased by 20% to 17 during the pandemic, and more than doubled to 29 during a busy day during the pandemic. This supports the assertion that communications had generally increased, exemplified in Table 1, where we see an increase reported by 70% of respondents against a reduced volume reported by 18%. This 70% figure was stable over the first and last groups of almost a thousand participants each and reduced to 68% in the middle cohort.

**Table 1:** Survey Section 1.

Baseline characteristics	Total group 1–30 days	Per cent	Cohort 1 1–12 days	Per cent	Cohort 2 13–20 days	Per cent	Cohort 3 21–30 days	Per cent
Participants	3047	100.0	891	29.2	987	32.4	1169	38.4
Survey start date		21 May 2020		21 May 2020		3 June 2020		10 June 2020
Survey end date		19 June 2020		2 June 2020		9 June 2020		19 June 2020
Average per day	102	30 days	69	12 days	141	7 days	117	10 days
Highest per day	368	10 June 2020	213	2 June 2020	278	3 June 2020	319	10 June 2020
Lowest per day	7	21 May 2020	4	24 May 2020	7	6 June 2020	6	14 June 2020
Survey completion time								
Median (min)	12.7		11.9		12.8		13.0	
25th percentile (min)	9.6		9.1		9.9		9.9	
75% percentile (min)	17.5		16.9		18.2		17.8	
	**Number**	**Per cent**	**Number**	**Per cent**	**Number**	**Per cent**	**Number**	**Per cent**
Participants	3047	100.0	891		987		1169	
Gender	3047		891		987		1169	
Female	2592	85.1	746	83.7	854	86.5	992	84.9
Male	435	14.3	139	15.6	128	13.0	168	14.4
Prefer not to say	16	0.5	5	0.6	4	0.4	7	0.6
Prefer to self describe	4	0.1	1	0.1	1	0.1	2	0.2
Age range	3047		1093		1142		1376	
Not stated	24	0.8	6	0.5	10	0.9	8	0.6
18–30	411	13.5	114	10.4	160	14.0	137	10.0
31–40	679	22.3	196	17.9	228	20.0	255	18.5
41–50	828	27.2	254	23.2	262	22.9	312	22.7
51–60	911	29.9	256	23.4	277	24.3	378	27.5
61–80	194	6.4	65	5.9	50	4.4	79	5.7
Comms volume due to COVID-19								
Markedly reduced	212	6.96	66	7.41	66	6.69	80	6.84
Somewhat reduced	327	10.73	98	11.00	118	11.96	111	9.50
About the same	377	12.37	102	11.45	130	13.17	145	12.40
Somewhat increased	809	26.55	223	25.03	259	26.24	327	27.97
Markedly increased	1322	43.39	402	45.12	414	41.95	506	43.28
Baseline characteristics	**Total group 1–30 days**	**Per cent**	**Cohort 1 1–12 days**	**Per cent**	**Cohort 2 13–20 days**	**Per cent**	**Cohort 3 21–30 days**	**Per cent**
Same post	2845		689		987		1169	
Yes	1590	55.9	399	57.9	545	55.2	646	55.3
Yes remotely	702	24.7	148	21.5	235	23.8	319	27.3
No—redeployed	306	10.8	77	11.2	120	12.2	109	9.3
Other	247	8.7	65	9.4	87	8.8	95	8.1
Main work pattern	3043		889		987		1167	
Full time 9–5	907	29.8	251	28.2	336	34.0	320	27.4
Full time shifts	573	18.8	180	20.2	164	16.6	229	19.6
Office based	206	6.8	74	8.3	81	8.2	51	4.4
Working remotely	467	15.3	132	14.8	146	14.8	189	16.2
Part time	587	19.3	166	18.7	171	17.3	250	21.4
Other	303	10.0	86	9.7	89	9.0	128	11.0
Seeing COVID-19 patients: face to face	3002		876		974		1152	
Daily	392	13.1	126	14.4	115	11.8	151	13.1
3–4 days a week	276	9.2	107	12.2	67	6.9	102	8.9
1–2 days a week/occasionally	575	19.2	174	19.9	177	18.2	224	19.4
Office based only	636	21.2	188	21.5	252	25.9	196	17.0
Working remotely only	688	22.9	168	19.2	222	22.8	298	25.9
Other	435	14.5	113	12.9	141	14.5	181	15.7
Work location	3047		891		987		1169	
Academic unit	8	0.3	3	0.3	3	0.3	2	0.2
Acute secondary care hospital	609	20.0	234	26.3	206	20.9	169	14.5
Acute tertiary hospital	820	26.9	252	28.3	300	30.4	268	22.9
Ambulance services trust	18	0.6	2	0.2	8	0.8	8	0.7
Community health trust	404	13.3	79	8.9	88	8.9	237	20.3
GP Practice/Primary Care Hub	76	2.5	24	2.7	38	3.9	14	1.2
Mental health trust	259	8.5	33	3.7	92	9.3	134	11.5
Other—add text—thank you	310	10.2	113	12.7	89	9.0	108	9.2
Working remotely	543	17.8	151	16.9	163	16.5	229	19.6

More staff chose a higher category of email and WhatsApp ranges with each type of day, and clearly demonstrated an increase during COVID-19 relative to before the pandemic. In Fig. 3, we see that on a busy workday prior to the pandemic, 13% of staff estimated receiving and sending 11–30 work-related WhatsApp messages. This increased to 26% of staff during COVID-19. Whilst only 1% of staff estimated receiving and sending 51–100 work-related WhatsApp messages a day before COVID-19, we see this rise to 13% during the pandemic. Despite the differences due to COVID-19, there is a large range shift from a quieter to a busy day for the same staff. The majority of staff reported a somewhat (27%) or a marked (43%) increase in work communications due to COVID-19.

### Section 4: Communications, work-life balance, and need for governance framework

We asked questions regarding work communications and how manageable they are perceived to be, as well as the ability to switch off when they wanted. The questions in this section were basic as an attempt to keep the survey both accessible on a mobile phone, and with stems still easy to understand and follow. This was likely the reason for over 2766 respondents completing these questions near the end of the survey, with a median completion time of almost 13 min.

A total of 76% of staff reported that prior COVID-19 they felt able to manage work-related communications during their work time, with 18% struggling. During COVID-19, these responses varied at 61 and 31%, respectively. The majority of staff (57%) felt they could not switch off during the pandemic, compared to 36% prior to COVID-19. Over half of staff strongly agreed that work communications slowed down their main work during both periods—51% before, and 64% during COVID-19.

The main impact, however, was outside work. NHS staff have access to their NHS email via a web interface which can be accessed from any location, including away from their work desktops. Almost half (48%) of staff reported family and home time being taken up with work-related communications, during COVID-19, compared to 23% before the pandemic. Of the 14% strongly reporting intrusion of home life, 49% reported email volumes ranging from 51 to >250 emails on a busy day, compared to 15% not reporting any intrusion of home life. Additional disruption of leisure time is shown in Table 2. Notably, travel time was not disrupted—perhaps due to the lockdown making travel easier for some staff, as traffic was markedly reduced and restricted only to key workers. Interestingly, 63% of respondents reported never having exchanged email directly with patients both before and during the pandemic.

**Table 2:** Survey Section 4: impact of digital communications on work and work-life balance.

Statement relating to work communications	Staff	Strongly disagree (%)	Disagree (%)	Not sure (%)	Agree (%)	Strongly agree (%)
It’s manageable during work	2798	3	15	6	58	18
& During COVID-19	2994	10	21	8	47	14
I can switch off when i want to	2766	9	27	12	43	9
& During COVID-19	2963	20	37	12	24	6
It can slow down my main work	2770	5	32	12	43	8
& During COVID-19	2981	5	22	8	44	20
it takes up my home/family time	2789	22	46	8	19	4
& During COVID-19	2990	17	28	7	34	14
It takes up my leisure time	2783	24	49	8	16	3
& During COVID-19	2979	20	32	7	30	10
It takes up my travel time	2778	30	46	8	13	4
& During COVID-19	2985	36	45	8	8	4

**Fig. 4:**
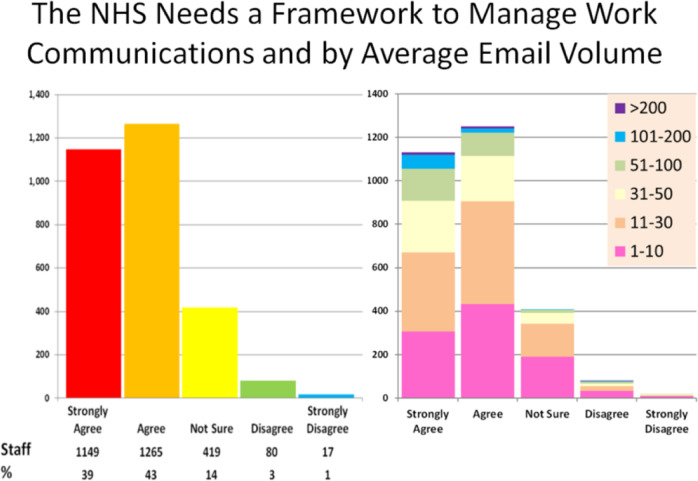
Figure pertaining to data collected from survey Section 4, showing respondent views on need for a governance framework relating to digital communications, further analysed by reported average email volume.

The majority (82%) of staff agreed that the NHS and other healthcare employers need guidance on actively managing the volume of work communication to improve staff wellbeing, with only 3% disagreeing and 14% unsure (Fig. 4). This agreement level was noteworthy, given the majority of those agreeing on a guidance framework had less than 30 emails a day.

## Discussion

### Summary of results

We demonstrated that work-related communication took up significant time for a large number of NHS staff before COVID-19, with 70% of respondents reporting an increase during COVID-19. Emails, phone calls and instant messaging applications not approved for medical use, in particular WhatsApp, were most frequently used. We have shown a 20% increase in the volume of email during COVID-19, with more than double the volume of email on busy days. We report 18% of staff unable to manage work communications during their work and 36% unable to switch off already before COVID-19, both adverse outcomes significantly exacerbated during COVID-19 to a staggering 31 and 57%, respectively.

We have shown some adverse consequences of this workload increase on both ‘main work’ and work-life balance, with 51% reporting distraction from main work and 23% reporting intrusion into home/family time before COVID-19, rising to 64 and 48% during COVID-19, respectively. Most staff (82%) stated they would welcome some form of guidance and a support framework to manage work communications reflects the widespread presence of this issue and the severity of its detrimental impacts on NHS staff and NHS core work.

### Context to NHS

In the context of the wider NHS, the primary consideration for staff is always patient care (DoHSC 2021). Chronic under-resourcing and excessive workload inevitably lead to prioritising patient care over other tasks, such as administration and work communications (House of Commons 2022). The large proportions of NHS staff who report unmanageable volume of work communications and their intrusion into travel, leisure and family/home time are symptomatic of this excessive workload, reflecting a lack of effective policy or supportive solutions from NHS employers to protect staff wellbeing.

With increased remote consultations (Greenhalgh et al. 2020), work from home or non-patient facing sites, email traffic and phone calls increase in volume during COVID-19 is a trend likely to continue beyond the pandemic, as remote working becomes a mainstay of clinical delivery (Murphy et al. 2021). Of the two types of communication, only email has an existing audit trail that can be incorporated into medical records, be in electronic or paper based. Therefore, the use of email for the purpose of exchanging clinical information, such as making patient referrals or providing clinical opinion, is likely to continue, complementing the use of electronic health record systems, such as those provided by EPIC and Cerner. Our finding that WhatsApp was the third most frequently used communication platform during COVID-19, despites its lack of governance framework in the NHS, shows that functionalities provided by instant message applications, in particular WhatsApp, have not been replaced by technologies approved for medical use, such as Microsoft Teams, Hospify or Panda.

### National extrapolation of email volume

We calculated that if we used the lower range value limit for each category of email responses, with the exception of the ‘1–10’ category being given a value of 5, for an average day, prior to COVID-19, with the proportion of staff in each category upscaled to 1,221,204 inboxes, allowing for 20% of staff working as part time at 33% of a full-time worker’s duration and assuming 20 working days a month, allowing within that for 25 working days annual leave a year, then the estimate of monthly email volume would be 329,824,163 emails per month. A Freedom of Information request to NHS Digital provided the volume of emails in November 2019 as 323,100,000 emails. We feel that the closeness of these values provides some external independent validation of our own volume results. Our volume projection is higher; this is likely due to participation bias as our respondents are more likely to engage with invitations to participate in research and respond to emails.

### Comparison with previous studies

The impeding mental, emotional and physical risks to NHS staff were recognised from the outset of the pandemic, together with the need to provide psychosocial support (Willian et al. 2020; Royal College of Physicians 2020). Multiple surveys investigating the psychological impact of the pandemic response in healthcare workers were conducted during COVID-19. Although these studies did not examine stress factors associated with patterns and volume of work communications and their impact on staff wellbeing and work-life balance, similarly to our study they report the widespread presence of workplace stress factors and their adverse impact on staff during COVID-19 (Gilleen et al. 2021; Rathod et al. 2020; Johnson et al. 2021), exacerbating adverse trends already present before the pandemic (GMC 2020; Sharp 2019).

The paucity of research focus concerning the impact of workplace communication in healthcare systems precedes the pandemic. Only a few small-scale studies exist for comparisons. Bittar and Nicholas (2018) surveyed 14 dermatologists and compared findings with the number of messages received in their electronic health record, showing that increased messaging, higher perceived burden, and longer time to completion were all correlated with the increased risk of burnout. However, this was a small study in one healthcare organisation. There is also evidence to suggest that electronic health records are independently associated with burnout (Alexander and Ballou 2018) but no volume data are presented.

In line with previous studies reporting on the vital role of WhatsApp in managing healthcare emergencies, including the Westminster Bridge attack, Manchester Arena bombing and the London Bridge attack (Skryabina et al. 2021), we confirm the equally vital role of WhatsApp in managing the ‘unfolding’ COVID-19 crisis, which was far more ‘complex and varied’ compared to the previous ‘exploding crises’ (Hutchings et al. 2021). Beyond the use of WhatsApp, there are no previous studies exploring communication pattern interplay with healthcare crises such as the COVID-19 pandemic (Greenberg et al. 2020; Horton et al. 2021).

Looking outside of healthcare, Barber et al. (2019) surveyed 254 people and showed that telepressure, or the ‘preoccupation and urge to respond quickly to message-based communications’ was associated with negative employee perceptions of work-life balance. In a work environment omnipresent with technological devices and their frequent use by others, employees may feel pressured or expected to do the same and be more responsive (MacCormick et al. 2012). Furthermore, employees experiencing high workplace telepressure have difficulty winding down during the evening since they are constantly on alert to incoming messages (Barber and Jenkins 2014; Van Laethem et al. 2018). Whilst our research did not directly measure burnout or the psychological impact of work communications on staff, our findings concerning the high perceived inability of staff to manage work communications during work hours, inability to switch off, and intrusion into home/family time (all of which are recognised drivers and symptoms of burnout) are consistent with the patterns observed in the wider telepressure literature, as well as the limited healthcare specific research.

### NHS employers’ duty of care

The trend towards remote working for NHS staff with little previous experience of working from home and the current adverse impact of work communications on staff as demonstrated by our finding bring call for NHS employers to consider providing an effective framework and support to staff to minimise stressors associated with work communications as part of their duty of care to protect staff health and wellbeing (Hodder 2020). Sectors outside of healthcare have already taken steps to mitigate these issues, such as the European legislations which promote a culture of not accessing emails whilst on leave (Eurofound 2021), to trends such as ‘Inbox Zero’ (French 2014). As a result, Adobe’s annual email usage survey (Adobe 2019) shows that time spent reviewing emails has been reducing since 2016, with an increase in the proportion of employees not checking emails before arriving at work or whilst on holiday.

Our study suggests that workplace communications across the healthcare sector spending time reviewing or managing communications outside work, had similar negative associations on work-life balance and this may inevitably contribute to staff burnout (Barber et al. 2019). Despite being seen on one hand as a convenient coping strategy to balance work and home life, ‘telework’ or managing work through digital communications from home, has brought blurred boundaries and work-life conflict, by transforming the temporal and spatial conditions of daily work and life routines (Thulin and Vilhelmson 2021). As a result, when applied to the entirety of the NHS, this implies that further work is needed to investigate and mitigate these negative effects of digital communication and remote working (Mellner 2016), and indeed, staff perceive this too in their clear desire for guidance on actively managing the volume of work communication to improve staff wellbeing.

### Limitations

A limitation of this study is its reliance on the self-reported data; this might be subject to bias on account of respondents’ inaccurate assessments during and before COVID-19. There is also a risk of sampling bias due to self-selection, as staff may have felt less inclined to take part in the study if they do not feel that they receive much work-related communication, potentially exaggerating the results. The comparison of the total estimated NHS email volume based on our extrapolated data against the actual NHS Digital data aim suggest that this bias has not significantly affected the validity of our results. While the survey links were available to all NHS staff via the UKRD forum website, most staff received the study invite via their employer. Therefore, further sampling bias occurred due to the speed of permissions in different NHS organisations. Consequently, staff were geographically represented across much of England, but Wales, Scotland, Ireland are underrepresented at present.

A total of 16% of those who opened the survey (564 staff) did not provide consent, did not confirm an NHS email address or left several sections unanswered; these responses were removed prior to data analysis. The study was open to responses between 21 May and 19 June 2020, that is approximately a month after the COVID-19 admission rates peaked in early to mid-April. Given the extreme clinical pressures, we feel that any earlier completion would not have been feasible. The weekend of 30/31 May was the first opportunity for many staff to focus on non-essential work, as also demonstrated by patterns in survey response rates. The timing of the survey being between the start of COVID-19 and the lifting of the lockdown was therefore optimal.

The survey size was limited by the conditions of the free survey licence as well the feasibility considerations pertaining to the time burden on the respondents during the pandemic. We have therefore focussed on the key survey aims, that is communications volume, platforms used and impact on core work and staff wellbeing. We have not examined perspectives, such as types of emails received, the drivers of staff engagement with work communications outside of work hours, or any measures of burnout. Future research is needed to explore these perspectives to provide broader insights and how results differ by work roles and patterns.

## Conclusions and recommendations

We have provided the first ever data concerning the communication mode types and usage frequencies during COVID-19. We have reported on the volume of traffic in the form of emails and WhatsApp messages. Our national projection is close to the actual reported number of emails obtained from NHS Digital through a Freedom of Information request. We have evaluated the change in communication patterns due to COVID-19 with retrospective and current evaluation and reported on staff work-life balance and confidence in managing communication.

Work-related digital communication takes up a significant amount of staff time and represents a considerable workload. Whilst there are useful aspects to this, digital communication has led to adverse effects for many staff as their main work has been disrupted and their home-life balance impacted. These negative effects have been further exacerbated during COVID-19 which saw an increased volume of communications. Staff support for a national policy and framework was captured in our survey, urgently calling on NHS employers to formulate guidance and protocols for managing this workload.

We provide recommendations to mobilise the healthcare sector with actionable steps to better understand and address adverse impacts arising from work-related communication overload (Table 3), including recognising communication overload as an area of clinical risk, commissioning and delivering further research, and providing dedicated staff support infrastructure at both the national and local levels.

**Table 3:** Recommendations.

High-level recommendation proposals to consider
1. The term ‘digital communication overload’ is separated from the term ‘information overload’ and is considered as a clinical risk within healthcare organisations.
2. The annual NHS staff survey is modified to include digital communication workloads and impact on work-life balance to confirm some of our results.
3. A national project is undertaken by NHS England (via NHS Digital) into digital communication volumes to understand variances and patterns in more detail and identify staff at risk of digital communications overload and potentially early signs of burnout.
4. The CQC, King’s Fund and the Faculty of Clinical Informatics are tasked to independently review correlations between NHS centres of excellence for communications culture and technology and patient outcomes and staff wellbeing and retention.
5. An NHS taskforce is convened to look at good practice around work-related communications and designing a training package to support staff to manage communication workload and produce guidance for employers on responsibilities in this area.
6. An NHS communication helpline for staff with excessive volumes of phone calls, emails and messages, is set up to provide immediate support. There may need ot be diversions or restriction of communications or a respite period with a support member from NHS Digital provided to be responsible for, oversee and support the affected user demonstrating stress and burnout.
7. The NIHR, NHSD, UKRD and R&D Forum are tasked with supporting research into work communication as a means of improving the lives of patients, relatives and healthcare professionals in line with Domains 4 and 5 of the NHS long term plan.
8. A National Director of Communications and Clinical Impact and Effectiveness is appointed within NHS to guide strategy, policy and research within the NHS to provide the basis of a national programme of work to coordinate and revolutionise communications innovation and to review the best use of newer technologies available to address these issues. An alternative would be for the NHS CRIO, CCIO and CMIO to lead this work.
9. The Chief Information Officers network is tasked with further insights into this work to confirm the problem, rally those willing to pilot new solutions and to support technology innovation projects to address these issues, disperse solutions and monitor milestones for research pilots.
10. The NHS wellbeing framework includes information on communications workload and staff management of this work as a measure to be reduced to allow more human-human interaction and to limit intrusion of home life and leisure time.

Reducing the risk of staff burnout is a priority for all healthcare systems worldwide (Wise 2022); thus, there is great scope to expand our insights through further research and policy action. Having reported on the hidden burden of clinical workload responsible for staff stress, seen to expand and worsen during crises such as COVID-19, we highlight the need to take appropriate actions in order to protect staff wellbeing and empower focus on core work: delivering patient care.

## Data Availability

Full access to the datasets generated and analysed during the present study are available upon reasonable request from the corresponding author.

## References

[CR1] Adobe (2019) 2019 Adobe Email Usage Study: Email Usage – Working Age Knowledge Workers (US Trended Results). https://www.slideshare.net/adobe/2019-adobe-email-usage-study. Accessed 6 Jan 2022

[CR2] Alexander AG, Ballou KA (2018) Work-life balance, burnout, and the electronic health record. Am J Med 857–858. 10.1016/j.amjmed.2018.02.033 10.1016/j.amjmed.2018.02.033 29608876

[CR3] Barber LK, Conlin AL, Santuzzi AM (2019). Workplace telepressure and work-life balance outcomes: the role of work recovery experiences. Stress Health.

[CR4] Barber LK, Jenkins JS (2014). Creating technological boundaries to protect bedtime: examining work-home boundary management, psychological detachment and sleep. Stress Health.

[CR70] Becker WJ, Belkin LY, Conroy SA, Tuskey S (2021) Killing Me Softly: Organizational E-mail Monitoring Expectations’ Impact on Employee and Significant Other Well-Being. J Manag 47(4):1024–1052. 10.1177/0149206319890655

[CR5] Belkin LY, Becker WJ, Conroy SA (2020). The invisible leash: the impact of organizational expectations for email monitoring after-hours on employee resources, well-being, and turnover intention. Group Organ Manag.

[CR6] Bittar PG, Nicholas MW (2018). The burden of inbox-messaging systems and its effect on work-life balance in dermatology. J Am Acad Dermatol.

[CR7] Blake H, Gupta A, Javed M (2021). COVID-Well Study: qualitative evaluation of supported wellbeing centres and psychological first aid for healthcare workers during the COVID-19 pandemic. Int J Environ Res Public Health.

[CR8] Dettmers J, Vahle-Hinz T, Bamberg E (2016). Extended work availability and its relation with start-of-day mood and cortisol. J Occup Health Psychol.

[CR9] DoHSC (Department of Health & Social Care) (2021) The NHS Constitution for England. https://www.gov.uk/government/publications/the-nhs-constitution-for-england/the-nhs-constitution-for-england#nhs-values. Accessed 30 Sept 2022

[CR10] Eurofound (2021) Right to disconnect: exploring company practices. https://www.eurofound.europa.eu/publications/report/2021/right-to-disconnect-exploring-company-practices. Accessed 30 Sept 2022

[CR11] French MK (2014) ‘Achieving “Inbox Zero”’. https://www.journalofaccountancy.com/issues/2014/jun/inbox-zero.html. Accessed 6 Jan 2022

[CR69] Gadeyne N, Verbruggen M, Delanoeije J, De Cooman R (2018) All wired all tired? Work-related ICT-us e outside work hours and work-to-home conflict: The role of integration preference integration norms and work demands. J Voc Behav 10786–99 S0001879118300381. 10.1016/j.jvb.2018.03.008

[CR12] General Medical Council (2019) Domain 3: communication partnership and teamwork. https://www.gmc-uk.org/ethical-guidance/ethical-guidance-for-doctors/good-medical-practice/domain-3---communication-partnership-and-teamwork. Accessed 3 Jan 2022

[CR13] General Medical Council (2020) National Training Survey 2020: summary of results. https://www.gmc-uk.org/-/media/documents/nts-results-2020---summary-report_pdf-84390984.pdf. Accessed 15 Sept 2022

[CR14] Gilleen J, Santaolalla A, Valdearenas L, Salice C, Fusté M (2021). Impact of the COVID-19 pandemic on the mental health and well-being of UK healthcare workers. BJPsych Open.

[CR15] Greenberg N, Docherty M, Gnanapragasam S (2020). Managing mental health challenges faced by healthcare workers during COVID-19 pandemic. BMJ.

[CR16] Greenhalgh H, Wherton J, Shaw S, Morrison C (2020). Video consultations for covid-19. BMJ.

[CR18] Hodder A (2020). New technology, work and employment in the era of COVID-19: reflecting on legacies of research. New Technol Work Employ.

[CR19] Horton T, Hardie T, Mahadeva S, Warburton W (2021) Securing a positive health care technology legacy from COVID-19. The Health Foundation. https://www.health.org.uk/publications/long-reads/securing-a-positive-health-care-technology-legacy-from-covid-19. Accessed 5 Jan 2022

[CR20] House of Commons (2022) https://committees.parliament.uk/publications/6158/documents/68766/default/. Accessed 15 Sept 2022

[CR21] Hughes O (2020) Microsoft Teams to be rolled out across NHS in response to coronavirus. Available via Digital Health. https://www.digitalhealth.net/2020/03/microsoft-teams-nhs-coronavirus/. Accessed 5 Jan 2022

[CR22] Hutchings R (2020) The impact of COVID-19 on the use of digital technology in the NHS. In: Briefing, Nuffield Health. Available via Nuffield Trust. https://www.nuffieldtrust.org.uk/files/2020-08/the-impact-of-covid-19-on-the-use-of-digital-technology-in-the-nhs-web-2.pdf. Accessed 5 Jan 2022

[CR23] Hutchings SD, Perry J, Park CL (2021) Command, control and communication (C3) during the COVID-19 pandemic; adapting a military framework to crisis response in a tertiary UK critical care centre. J Intensive Care Soc 23(2). 10.1177/1751143720982191 10.1177/1751143720982191 PMC9125435 35615232

[CR24] Iliffe S (2008). Myths and realities in multidisciplinary team-working. London J Prim Care (Abingdon).

[CR25] Jisc Online Surveys (2022) https://www.onlinesurveys.ac.uk/. Accessed 5 Jan 2022

[CR26] Johnson S, Dalton-Locke C, San Juan NV (2021). Impact on mental health care and on mental health service users of the COVID-19 pandemic: a mixed methods survey of UK mental health care staff. Soc Psychiatry Psychiatr Epidemiol.

[CR28] Van Laethem M, van Vianen AEM, Derks D (2018) Daily fluctuations in smartphone use, psycholgical detatchment, and work engagement: the role of workplace telepressure. Front Psychol 9. 10.3389/fpsyg.2018.01808 10.3389/fpsyg.2018.01808 PMC6166396 30319504

[CR68] Marsh E, Vallejos EP, Spence A (2022) The digital workplace and its dark side: An integrative review. Comput Human Behavior. 128107118–S0747563221004416. 10.1016/j.chb.2021.107118

[CR29] MacCormick JS, Dery K, Kolb DG (2012). Engaged or just connected? Smartphones and employee engagement. Organ Dyn.

[CR30] Mellner C (2016). After-hours availability expectations, work-related smartphone use during leisure, and psychological detachment: the moderating role of boundary control. Int J Work Heal Manag.

[CR31] Morris C, Scott RE, Mars M (2021). WhatsApp in clinical practice – the challenges of record keeping and storage. A scoping review. Int J Environ Res Public Health.

[CR32] Murphy M, Scott LJ, Salisbury C, Turner A, Scott A, Denholm R, Lewis R, Iyer G, Macleod J, Horwood J (2021). Implementation of remote consulting in UK primary care following the COVID-19 pandemic: a mixed-methods longitudinal study. Br J Gen Pract.

[CR33] NHS Digital (2020) Freedom of Information Act (FOIA) 2000 ref: NIC-386876-Z6W6W. Internal document available on request

[CR34] NHS Digital (2022) National Workforce Data Set (NWD) guidance documents (Appendix A Staff Group Definitions v10.0) https://digital.nhs.uk/binaries/content/assets/website-assets/data-and-information/areas-of-interest/workforce/appendix-a-staff-group-definitions-v10.0_final.docx. Accessed 30 Sept 2022

[CR35] NHS England (2021) https://www.england.nhs.uk/2021/03/nhs-celebrates-the-vital-role-hundreds-of-thousands-of-women-have-played-in-the-pandemic/. Accessed 10 Aug 2022

[CR36] NHS England (2022) Using mobile messaging. https://transform.england.nhs.uk/information-governance/guidance/use-mobile-messaging-software-health-and-care-settings/. Accessed 3 Oct 2022

[CR38] Nikolic A, Wickramasinghe N, Clayton-Platt D, Balakrishnan V, Smart P (2018). The use of communication apps by medical staff in the Australian health care system: survey study on prevalence and use. JMIR Med Inform.

[CR39] Nohammer E, Stichlberger S (2019). Digitalization, innovative work behavior and extended availability. J Bus Econ.

[CR40] Patel RS, Bachu R, Adikey A, Malik M, Shah M (2018). Factors related to physician burnout and its consequences: a review. Behav Sci (Basel).

[CR67] Puranik H, Koopman J, Vough HC (2020) Pardon the Interruption: An Integrative Review and Future Research Agenda for Research on Work Interruptions. J Manag 46(6):806–842. 10.1177/0149206319887428

[CR41] Qualtrics XM (2022) https://www.qualtrics.com/uk/. Accessed 5 Jan 2022

[CR43] Rathod S (2020). Psychological impact of COVID-19 pandemic: protocol and results of first three weeks from an international cross-section survey-focus on health professionals. J Affect Disord Rep.

[CR44] Royal College of Physicians (2020) COVID-19 and its impact on NHS workforce. https://www.rcplondon.ac.uk/news/covid-19-and-its-impact-nhs-workforce. Accessed 5 Jan 2022

[CR45] Sharp EW (2019) Burnout in NHS Surgeons: a systematic review and narrative synthesis of risk-factors, effects, interventions and implications for stakeholders. Brighton and Sussex Medical School. http://sro.sussex.ac.uk/id/eprint/88269/. Accessed 6 Jan 2022

[CR46] Skryabina E, Betts N, Reedy G (2021). UK healthcare staff experiences and perceptions of a mass casualty terrorist incident response: a mixed-methods study. Emer Med J.

[CR47] Stevens S, Pritchard A (2020) Urgent next steps on NHS response to COVID-19. In: Next steps on NHS response to COVID-19. NHS England and NHS Improvement. Available via NHS England. https://www.england.nhs.uk/coronavirus/wp-content/uploads/sites/52/2020/03/urgent-next-steps-on-nhs-response-to-covid-19-letter-simon-stevens.pdf. Accessed 5 Jan 2022

[CR65] Stich JF, Tarafdar M, Cooper CL (2018) Electronic communication in the workplace: boon or bane?. J Organ Eff: People Perform 5(1):98–106. 10.1108/JOEPP-05-2017-0046

[CR66] Stich JF, Tarafdar M, Stacey P, Cooper CL (2019) E-mail load workload stress and desired e-mail load: a cybernetic approa ch. Information Technology & People 32(2):430–452. 10.1108/ITP-10-2017-0321

[CR64] Tarafdar M, Tu Q, Ragu-Nathan TS (2014) Impact of Technostress on End-User Satisfaction and Performance. Journal of Management Information Systems 27(3):303–334. 10.2753/MIS0742-1222270311

[CR48] The King’s Fund (2019) Digital health care: our position. https://www.kingsfund.org.uk/projects/positions/digital-health-care. Accessed 3 Jan 2022

[CR49] Thomas K (2018). Wanted: a WhatsApp alternative for clinicians. BMJ.

[CR50] Thulin E, Vilhelmson B (2021) Pacesetters in contemporary telework: how smartphones and mediated presence reshape the time–space rhythms of daily work. New Technol Work Employ 1–20. 10.1111/ntwe.12224

[CR52] Wang MJ, Low A (2019). Chinese employees suffer from telepressure: how can employees contribute to solutions. Clin Med Images.

[CR63] Waldhauser B (2019) ICT-related interruptions and work-related stress: a systematic literature review. Cryptocurrencies, Technostress, HR Management, Didactics and Group Reflection, 27:67–78.

[CR53] Willian J, King AJ, Jeffrey K (2020). Challenges for NHS hospitals during COVID-19 epidemic. BMJ.

[CR54] Wise J (2022). Burnout among trainees is at all time high, GMC survey shows. BMJ.

[CR55] World Health Organisation (2020) WHO Director-General’s statement on IHR Emergency Committee on Novel Coronavirus (2019-nCoV). https://www.who.int/director-general/speeches/detail/who-director-general-s-statement-on-ihr-emergency-committee-on-novel-coronavirus-(2019-ncov). Accessed 5 Jan 2022

